# Supplementary material for: Immunoprofiling Identifies Functional B and T Cell Subsets Induced by an Attenuated Whole Parasite Malaria Vaccine as Correlates of Sterile Immunity
Source: Vaccines (Basel). 2022 Jan 14;10(1):124. doi: 10.3390/vaccines10010124 (PMC8780163; doi:10.3390/vaccines10010124)

Supplementary Data

Mura et al. Immunoprofiling Identifies Functional B and T Cell Subsets Induced by an Attenuated Whole Parasite Malaria Vaccine as Correlates of Sterile Immunity

Supplementary Table S1: Immune measures (out of 1,316 measures) that were modified after immunization (T1) from baseline (T0) (p value < 0.05, q value < 0.2), with 26 having a q value < 0.05 and defined as immunization signature. The names of the immune measures appears as immunemeasure.stimulant.timepoint for cellular assays and readout.specificity.timepoint for serological assays.

	Immune measures	p value	q value
1	BC.SPZ.T1	3,81E-06	1,16E-03
2	Naïve_BC.SPZ.T1	1,13E-05	1,72E-03
3	B ELIspot.CSP.T1	6,10E-05	3,71E-03
4	MBC.SPZ.T1	5,93E-05	3,71E-03
5	IgD+MBC.SPZ.T1	4,19E-05	3,71E-03
6	IgD-MBC.SPZ.T1	1,45E-04	7,33E-03
7	IFA.SPZ.T1	3,29E-04	1,43E-02
8	B ELIspot.SPZ.T1	4,72E-04	1,48E-02
9	ELISA.CSP_FL.T1	4,81E-04	1,48E-02
10	T CD4+CXCR3-CCR6-TNFa+IFNg+.SPZ.T1	4,88E-04	1,48E-02
11	MSD.CSP_NANP.T1	9,77E-04	2,47E-02
12	T CD4+CXCR3+TNFa+IFNg+.CSP.T1	9,77E-04	2,47E-02
13	B ELIspot.MSP.T1	1,12E-03	2,58E-02
14	T CD4+CXCR3-CCR6-TNFa+.SPZ.T1	1,34E-03	2,58E-02
15	lymphocytes.SPZ.T1	1,36E-03	2,58E-02
16	CD38hiCD24hi_BC.SPZ.T1	1,23E-03	2,58E-02
17	ELISA.AMA1.T1	1,66E-03	2,97E-02
18	lymphocytes.CSP.T1	1,86E-03	3,09E-02
19	lymphocytes.M.T1	1,93E-03	3,09E-02
20	IL8.AMA1.T1	2,49E-03	3,29E-02
21	cTfh1_TNFa+.M.T1	2,44E-03	3,29E-02
22	T CD4+CXCR3+TNFa+.SPZ.T1	2,44E-03	3,29E-02
23	T CD4+TNFa+IFNg+.CSP.T1	2,44E-03	3,29E-02
24	B ELIspot.AMA.T1	2,69E-03	3,40E-02
25	cTfhCXCR3+CCR6+.CSP.T1	3,36E-03	4,00E-02
26	T CD4+TNFa+IFNg+.CSP.T1	3,42E-03	4,00E-02
27	IL8.CelTOS.T1	4,88E-03	5,50E-02
28	T CD4+TNFa+.SPZ.T1	5,40E-03	5,86E-02
29	B ELIspot.CelTOS.T1	6,12E-03	6,41E-02
30	T CD4+CXCR3+TNFa+IFNg+.SPZ.T1	6,84E-03	6,93E-02
31	T CD4+CXCR3+CCR6+.CSP.T1	8,47E-03	8,31E-02
32	cTfh2.CSP.T1	9,83E-03	8,54E-02
33	T CD4+CXCR3-CCR6-IFNg+.CSP.T1	9,28E-03	8,54E-02
34	T CD8+CXCR3+TNFa+.M.T1	9,28E-03	8,54E-02
35	T CD8.M.T1	9,83E-03	8,54E-02
36	IFNg.lSPZ.T1	1,07E-02	9,06E-02
37	T CD8+CXCR3-CCR6-TNFa+.CSP.T1	1,15E-02	9,22E-02

38	T CD8+CXCR3+.CSP.T1	1,14E-02	9,22E-02
39	T CD8+.SPZ.T1	1,33E-02	1,03E-01
40	IL13.CSP.T1	1,41E-02	1,07E-01
41	IL6.CSP.T1	1,61E-02	1,19E-01
42	IL4.CSP.T1	1,92E-02	1,26E-01
43	IL4.hSPZ.T1	1,99E-02	1,26E-01
44	cTfh2IFNg+.M.T1	1,85E-02	1,26E-01
45	T CD3+.M.T1	1,88E-02	1,26E-01
46	T CD8+CCR6+TNFa+.CSP.T1	1,85E-02	1,26E-01
47	T CD8+CXCR3-CCR6-.CSP.T1	1,97E-02	1,26E-01
48	T CD8+CXCR3+CCR6+IFNg+.CSP.T1	1,85E-02	1,26E-01
49	T CD4+CXCR3+CCR6+IFNg+.M.T1	2,10E-02	1,28E-01
50	T CD8+CCR6+IFNg+.CSP.T1	2,10E-02	1,28E-01
51	IL13.CelTOS.T1	2,22E-02	1,32E-01
52	IL13.hSPZ.T1	2,32E-02	1,34E-01
53	cTfh2IFNg+.CSP.T1	2,34E-02	1,34E-01
54	cTfhCXCR3+CCR6+IFNg+.M.T1	2,69E-02	1,46E-01
55	T CD4+CXCR3+IFNg+.CSP.T1	2,69E-02	1,46E-01
56	T CD4+CXCR3-CCR6-IFNg+.M.T1	2,69E-02	1,46E-01
57	cTfh.SPZ.T1	2,74E-02	1,46E-01
58	cTfr.M.T1	3,02E-02	1,58E-01
59	T CD4+CCR6+IFNg+.M.T1	3,42E-02	1,73E-01
60	T CD4+TNFa+IFNg+.SPZ.T1	3,42E-02	1,73E-01
61	IL12.CelTOS.T1	3,60E-02	1,80E-01

BC = B cells ; MBC = memory B cells ; T = T lymphocytes ; cTfh = circulating follicular helper T cells ; cTfr = circulating follicular regulatory T cells ; hSPZ/lSPZ = high/low dose of sporozoites.

Supplementary Table S2: Antibodies used for B cell and T cell flow cytometry panels.

B cell panel: antibodies	Provider	reference	dilution	clone
L/D fixable blue dead cell stain kit	ThermoFisher	L23105	1:250	-
CD19 PercP-Vio700	Miltenyi	130-113-733	1:100	LT19
CD20 APC-Vio770	Miltenyi	130-111-341	1:50	REA780
CD24-PE	Miltenyi	130-112-656	1:50	REA832
IgD-FITC	Miltenyi	130-124-213	1:50	IgD26
CD27-PE Vio770	Miltenyi	130-113-641	1:50	REA499
CD69-BV711	BD Biosciences	310944	1:10	FN50
CD38-APC	Miltenyi	130-123-852	1:100	REA671
CD3-Vioblue	Miltenyi	130-113-133	1:20	BW264/56

T cell panel: antibodies	Provider	reference	dilution	clone
L/D fixable blue dead cell stain kit	ThermoFisher	L23105	1:250	-
Anti-biotin microbeads UP	Miltenyi	130-105-637	1:5	-
CD69-biotin	Miltenyi	130-112-609	1:50	REA824
CD8 FITC	Miltenyi	130-110-509	1:50	REA715
Anti-biotin PE	Miltenyi	130-110-951	1:50	REA746
CXCR5 PEVio770	Miltenyi	130-117-358	1:50	REA103
CD3 Percp-Cy5.5	BD Biosciences	340949	1:5	SK7
CXCR3 BV711	BD Biosciences	563156	1:10	1C6/CXCR3
CCR6-APC R700	BD Biosciences	565173	1:50	11A9
CD4 APC H7	BD Biosciences	560158	1:50	RPA-T4
Foxp3 PE CF594	BD Biosciences	562421	1:20	259D/C7
TNFa V450	BD Biosciences	561311	1:20	MAb11
IFNg APC	BD Biosciences	551385	1:100	4SB3

Supplementary Figure S1: T cell gating strategy.

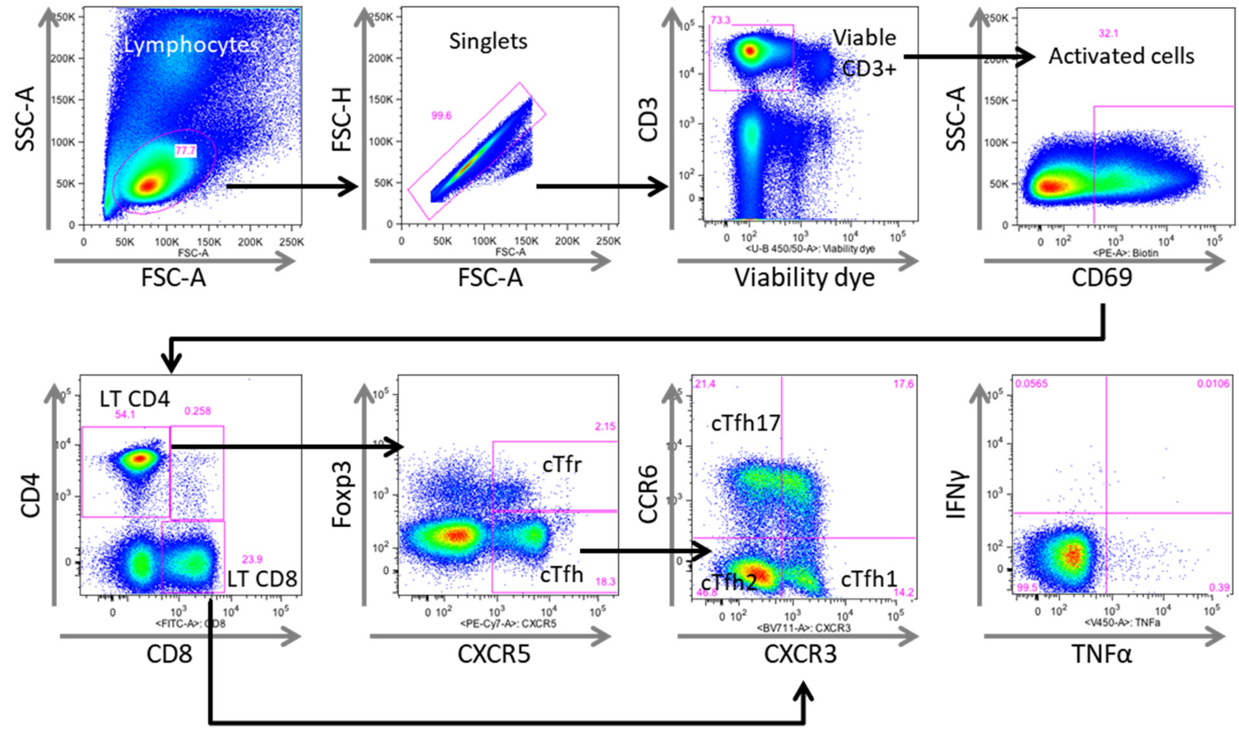

Supplementary Figure S2: B cell gating strategy.

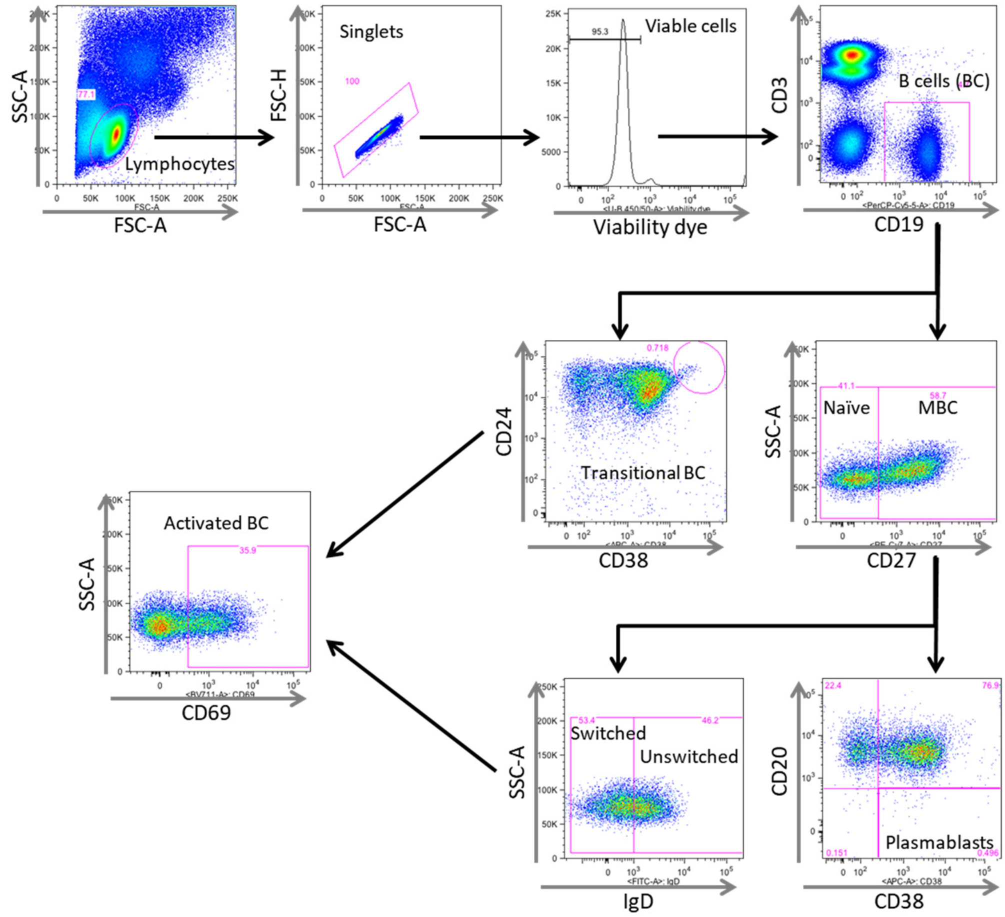

Supplementary Figure S3: Workflow of computational and statistical analysis

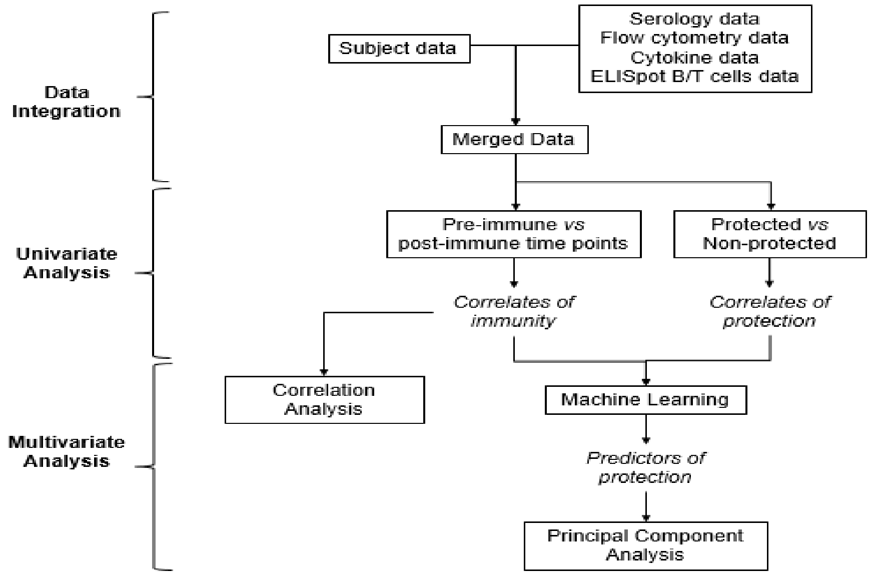

Supplementary Figure S4: Correlation matrices of cytokines pattern response measured in the supernatant of PBMCs at T1, after 48 hours of stimulation with AMA-1, CelTOS, CSP, SPZ at low (lSPZ) or high level (hSPZ) and TRAP. (A) Correlation matrices including only protected volunteers (n=8). (B) Correlation matrices including only non-protected volunteers (n=4). Only significant correlations (Spearman, ρ >0.4, p<0,05) are represented on the maps.

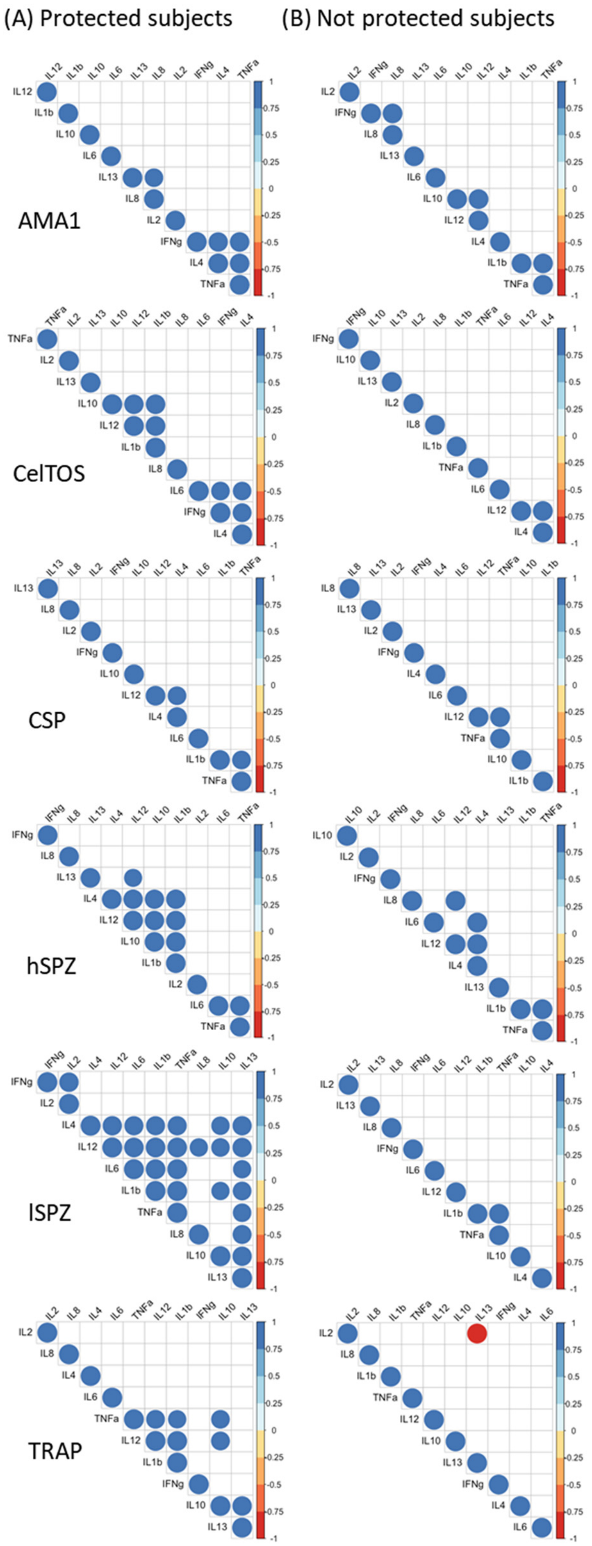

Supplement: Supplementary file 1 [file vaccines-10-00124-s001.zip › vaccines-1534528-supplementary.pdf]
